# A Concise Review on the Potential Applications of *Rugulopteryx okamurae* Macroalgae

**DOI:** 10.3390/md21010040

**Published:** 2023-01-03

**Authors:** Ligia Barcellos, Christopher K. Pham, Gui Menezes, Raúl Bettencourt, Nieta Rocha, Miguel Carvalho, Helena P. Felgueiras

**Affiliations:** 1 Centre for Textile Science and Technology (2C2T), University of Minho, Campus Azurém, 4800-058 Guimarães, Portugal; 2 Institute of Marine Sciences—OKEANOS, University of the Azores, 9901-862 Horta, Portugal; 3 Circular Blue Group, TERINOV—Science and Technology Park, Terceira Island, Terra Chã, 9700-702 Angra do Heroísmo, Portugal

**Keywords:** *Rugulopteryx okamurae*, invasive seaweed, main component or additive, applications

## Abstract

The brown macroalgae of the species *Rugulopteryx okamurae* has reached European waters and the Strait of Gibraltar as an invasive species. The proliferation and colonization of the species in subtidal and intertidal zones of these regions imposes significant threats to local ecosystems and additionally represents a significant socioeconomic burden related to the large amounts of biomass accumulated as waste. As a way to minimize the effects caused by the accumulation of algae biomass, investigations have been made to employ this biomass as a raw material in value-added products or technologies. The present review explores the potential uses of *R. okamurae*, focusing on its impact for biogas production, composting, bioplastic and pharmaceutical purposes, with potential anti-inflammatory, antibacterial and α-glucosity inhibitory activities being highlighted. Overall, this species appears to present many attributes, with remarkable potential for uses in several fields of research and in various industries.

## 1. Introduction

**Figure 1:**
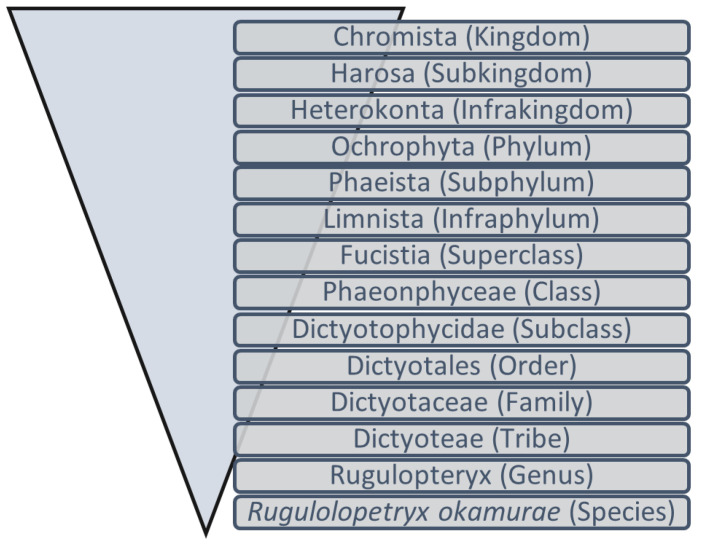
Schematic representation of the taxonomic position of Rugulopteryx okamurae [13].

Invasive non-native species are transported out of their natural habitats, by human hand, in areas not naturally reachable [1]. After these species overcome ecological barriers, they begin to settle in the region [2,3]. Space dominance occurs as native species are replaced, leading to environmental and/or socioeconomic impacts in the area [1]. Important factors are considered for the successful development of non-native species in new habitats, namely the biological characteristics of the non-native species, the quantity and frequency of their propagule introductions, and the susceptibility of the native species [2,3,4]. Non-native macroalgae invasion in new habitats is one of the main threats to marine biodiversity as it seriously impacts marine ecosystems. The observed negative effects are the alteration of competitive relationships and a decrease in ecosystem biodiversity in the invaded habitat [5]. In 2016, it was estimated that at least 364 non-native algae invasive species were distributed throughout main bioregions worldwide [6], including the species *Rugulopteryx okamurae* (Dictyotales, Ochrophyta) (Figure 1), the object of this study.

The brown macroalga *R. okamurae* is one of the most recently identified non-native invasive species. This invasive nature was detected in France, in the Strait of Gibraltar (Ceuta and Tarifa) and in the São Miguel Island (Azores) [7], together with other oceanic islands. The implications resulting from the massive invasion of this species are significant and involve several spheres. Indeed, this invasive species is threatening local ecosystems with the possibility of triggering a future irreversible situation. Actions to minimize the identified effects are urgent and may include different stakeholders. The multidisciplinary collaborations and integrated approaches are an essential way to mitigate the impacts [8]. Many specialists have reinforced the need to reformulate algae invasion as a multidisciplinary, problem-oriented discipline rather than a purely ecological concern [9]. Most studies on *R. okamurae* are oriented towards disseminating and monitoring the proliferation and impact of the invasive species and in identifying the causes behind its invasive character. Despite still being incipient, research on potential applications of the macroalgae is also being performed as a way of minimizing the accumulation of macroalgae residue and the costs associated with its removal and elimination from affected regions [10]. This research gives value to the residue [11], transforming a problem into an opportunity for obtaining new marketable products [12].

For centuries, macroalgae have been consumed by the populations of East Asia. As health benefits of algae incorporation within diets have been established, consumption in Western countries has also increased. Bioactive compounds (such as sulfated polysaccharides, polyphenols, pigments and carotenoids) found in algae are known to prevent the appearance of diseases, such as cancer, low blood pressure and hyperglycemia, and to protect the human body against foreign invaders by improving our antiviral, anti-inflammatory, immunomodulatory and neuroprotective defenses [14]. Many brown macroalgae are already being used as raw materials in everyday products; hence, the application of *R. okamurae* in such products has potential to be successful. This concise literature review provides a first look on the current uses of *R. okamurae*, as to encourage new products’ development and promote problem-solving opportunities for communities affected by invasive algae species, including the Strait of Gibraltar or in several islands of the Azores archipelago (large algae biomass accumulation).

## 2. *R. okamurae* Incidence in European Waters

**Figure 2:**
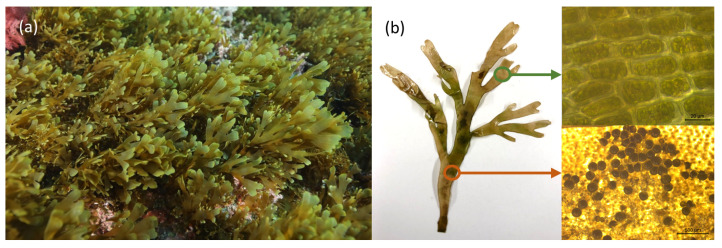
(**a**) *R. okamurae* brown macroalgae growing in Mediterranean Sea (adapted from [21], published by MDPI in 2021 with CC BY 4.0 permission). (**b**) Photograph of *R. okamurae* alga collected from the coast of São Miguel Island, with brightfield microscopic micrographs being taken to highlight details of the different regions of the alga.

The *R. okamurae* brown macroalgae (Figure 2) is a native species in temperate waters of the Northwest Pacific Ocean, in particular Japan and Korea [15]. This species was first reported outside its native habitat in the Mediterranean Sea in 2002, near a small port in Lagoa Thau (France), a well-known hotspot for marine species introduction in Europe [16]. Researchers believe the seaweed was introduced during the importation of the Japanese oyster culture *Crassostrea gigas* [17]. Considering that there is a frequent aquaculture transport between Thau Lagoon and other facilities, the risk of secondary dispersal of *R. okamurae* along the Mediterranean and southern North Atlantic coasts was inevitable [16,17,18]. In 2015, *R. okamurae* was reported for the first time on the south side of the Strait of Gibraltar, in Ceuta (North Africa) [19]. For a year, this macroalgae became a competitive invasive species with accelerated growth. The species extended over much of subtidal illuminated bottoms to a maximum perceived depth of 40 m, with greater coverage (80–90%) between depths of 10 and 20 m, replacing much of the native benthic communities. Colonization started on the south side of the Strait of Gibraltar, expanding geographically to the northern coastal zone, with a catastrophic propagation from 2015 to 2017. Afterwards, it extended to the south of the Iberian Peninsula, towards the Atlantic coast (2018) and the Mediterranean coast (2019) [20].

*R. okamurae* caused evident ecological impacts, namely biodiversity reduction, in the intertidal zone with the accumulation of substrates on illuminated rocky bottom areas and beaches, which also had serious implications for tourism and public health [20,22,23]. For this reason, more than 5000 tons of biomass had to be extracted from Ceuta beach [22,23] and the same has been occurring in some beaches of the Azores islands in recent years. Moreover, this invasion impacted the local fishing activity, since the algae becomes entangled in the nets, ropes and hooks, significantly reducing their fishing efficiency, with the fishing industry demanding a solution [23].

In terms of ecosystem impact, there have been indications that *R. okamurae* can also be established on partially shaded vertical surfaces and in cavities, posing a threat to the sessile fauna of these enclaves, including species sensitive to physicochemical changes [23].

The aggressive invasion by *R. okamurae* was observed for the first time in European waters, with a quick raise of its density and biomass. For instance, no other species of invasive algae showed such intensity of expansion in Spain. Currently, it is not possible to quantify the substrate, but it is possible to understand the extent of the invasion from the 5000 tons extracted from the island of Ceuta [23], along the summer of 2016 [22].

In Portugal, *R. okamurae* was detected near the main port of São Miguel Island, in Azores archipelagos (NE Atlantic), in 2019. It was initially identified as *Dictyota* spp., for which the phenotype is similar. It is likely the species introduced by waters of ballasts or through ship hulls coming from the Mediterranean community colonized in the Strait of Gibraltar. In subsequent years, there was a significant expansion and posterior dominance of the habitat in well-lit and shaded rocky surfaces of subtidal regions throughout the southern coast of São Miguel. Its invasive character was confirmed in the region with replacement of the native biota and an occupancy rate that reached almost 100% in some locations. Furthermore, in May 2021, algal substrate accumulation was reported on beaches of Faial Island [7]. The species appeared in São Miguel Island with the same accelerated colonization as in the Strait of Gibraltar, with intense proliferation and habitat domain [7,16]. In Azores, significant changes in the structure of shallow-water marine benthic communities have already been noticed, with a significant absence of diversification of native species, which ratifies the invasive character of this alga [7].

**Figure 3:**
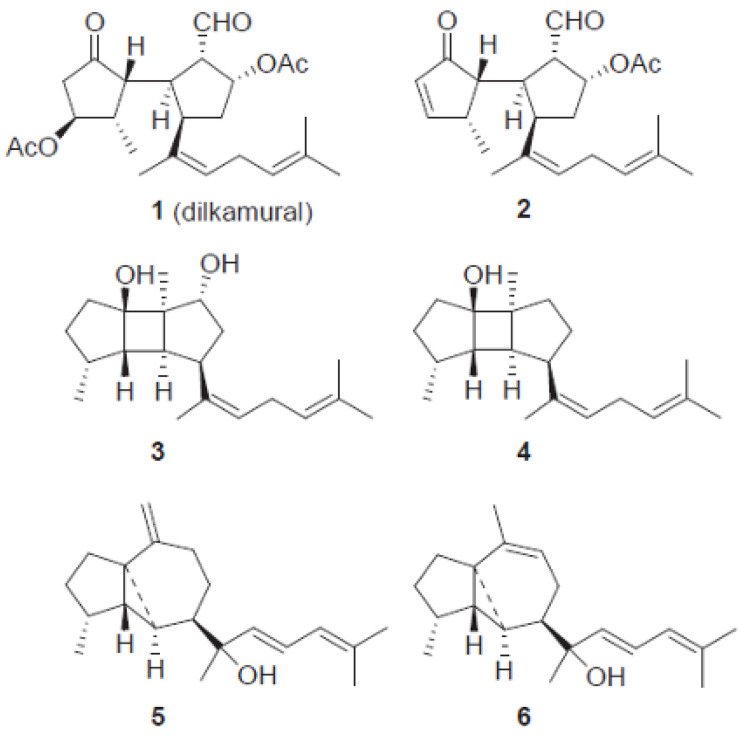
Chemical structures of the six secondary metabolites (compounds **1**–**6**) isolated from *R. okamurae* collected at the Strait of Gibraltar (from [24], published by Elsevier in 2021 with CC-BY-NC-ND permission).

The invasive success on the coasts of southwest Europe boosted research on the presence of the potential role of chemical defenses of *R. okamurae* that would justify the expansion of the macroalgae in these new habitats. Recently, a phytochemical study performed to identify the secondary metabolites of the invasive alga *R. okamurae* collected in the coasts of the Strait of Gibraltar determined the existence of six secondary metabolites (compounds **1**–**6**). Nuclear magnetic resonance (NMR) was used to establish the diterpenes chemical structures, as shown in Figure 3) [24].

A study established that, of these six isolated compounds, one of them, namely the dilkamural (compound **1**), may be responsible for the deterrent and toxic effect of the macroalgae against herbivores. This effect may be related to the high concentration of this metabolite compared to others found in *R. okamurae*. In Table 1, it is possible to see the high content of the dilkamural compound compared to other metabolites found. This investigation highlighted the high content in dilkamural compound (4.21 ± 0.39% of the dry weight of algae) by *R. okamurae*, which may be responsible for the low preference of native consumers for the invasive species [24].

It is noteworthy that previous chemical studies carried out on native specimens of *R. okamurae* collected in various locations along the Japanese coast had already shown the presence of secondary metabolites of the terpenoid class. However, compounds isolated from algae collected in the Strait of Gibraltar showed significant differences in concentrations from those found previously in native Japanese specimens [24]. The concentration of dilkamural found in native species was 1.9% (*w/w*) [24,25], less than half the average concentration recorded in the Strait of Gibraltar species study. However, this comparison is preliminary, since for a well-founded comparison, it is necessary to perform an updated quantitative analysis of dilkamural in *R. okamurae* species from its native range (Northwest Pacific Ocean) using the same biomass handling and analytical procedures employed in the research made with invasive species [24].

Since the results suggest a potential relationship between the concentration of dilkamural and the invasiveness of *R. okamurae*, there are two possible hypotheses that may support this affirmation and that still need to be deepened in future research [24]: (1) the lack of specialized herbivores in the new area (non-native area) may increase defense levels (e.g., toxins) against generalist herbivores in the new areas [24,26,27], and (2) the pressure endured from herbivores during the early stages of invasion may induce a positive selection towards *R. okamurae* (i.e., genotypes) with high concentrations of dilkamural being possible, which may cause the alga to establish and spread more rapidly [24,28].

## 3. Applications

Since ancient times, seaweeds have been used as food supplements, particularly in Asian countries. As science and technology evolved, more applications emerged, some of those using specific bioactive compounds extracted from the algae structure. Indeed, many seaweeds bioactive compounds have been reported to have activities that benefit human health [29]. Each bioactive compound has specific properties and biotechnological applications. Some bioactive compounds are already known, such as alginate (polysaccharide frequently used in the biomedical and food industries); phlorotannins; fucoidans; k-carrageenan; λ-carrageenan and agar; phenolic, flavonoid and alkaloid compounds; halogenated metabolites; diterpene; sesquiterpene; Griffith (protein); ulvan; and others [30]. Moreover, phycocolloids, extracted from brown and red algae, have been used in the food industry as gelling agents; in pharmaceutical formulations as dressings and drug coatings; for biotechnological purposes in culture media for many microorganisms; and in the cosmetic industry as body lotions, soaps, shampoos and toothpaste additives. Seaweeds have also been traditionally used in animal feed and agriculture and in biodiesel production [30]. To date, there are few reports on the prospective applications of *R. okamurae*. Most of those address its use as biomass for anaerobic co-digestion, composting and biofertilizer production, and the development of bio-based plastic materials (bioplastics). Additionally, potential anti-inflammatory and antimicrobial activities have been suggested, as well as the ability to inhibit α-glucosidase, which may be attractive features for the biomedical, pharmaceutical or food industries [12].

### 3.1. Applications Using R. okamurae Biomass as a Raw Material

#### 3.1.1. Anaerobic Digestion for Biogas Production

**Figure 4:**
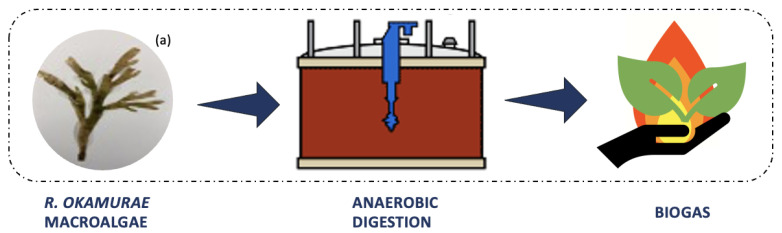
Anaerobic digestion for biogas production process using *Rugulopteryx okamurae* biomass. (**a**) Photograph of *Rugulopteryx okamurae* alga collected from the coast of São Miguel Island.

The use of *R. okamurae* biomass as a substrate in anaerobic digestion has emerged as a solution for the problem of accumulating tons of algal biomass along the coasts. Anaerobic digestion is a biological process (Figure 4) that occurs in the absence of oxygen, in which microorganisms metabolize organic matter, generating high calorific value biogas as a final product (mainly methane, 60–70%) [11]. Biogas is produced for energy purposes, as a renewable energy source. It can be obtained from wastes, thus contributing to a reduction in greenhouse gas emissions, as the consumption of fossil fuels and the amount of organic waste sent to landfills decrease [31].

Macroalgae have been used as organic matter for this biological process since it generates high methane yield coefficient. However, macroalgae cannot be processed on their own since, as a single substrate for biogas production, it may not have an ideal carbon/nitrogen ratio, resulting in a deficient anaerobic digestion process and low methane yield [11]. In anaerobic digestion, the ideal carbon/nitrogen ratio for sustaining optimal methane production must be between 25 and 30. With this ratio, it is possible to avoid the formation of inhibitory substances during processing [11,32]. As a single substrate, *R. okamurae* biomass presents a carbon/nitrogen ratio of 15.2, below the optimal range for successful anaerobic digestion [11]. Co-digestion with a mixture of different wastes has been proposed to improve the carbon/nitrogen ratio and, thus, the overall process [11,33,34]. For instance, *R. okamurae* (nitrogen-rich biomass) [11,35] can be combined with the semi-solid co-substrate generated from olive oil production process (OMSW), a carbon-rich substrate (ratio of 31.4). This combination allows for a balanced ratio, obtaining carbon/nitrogen values of 27.4, 24.9 and 23.3 in the proportions 1.0 *R. okamurae* to 3.0 OMSW, 1.0 *R. okamurae* to 1.5 OMSW, and 1.0 *R. okamurae* to 1.0 OMSW. Co-digestion guarantees an effective biodegradation of the organic matter, hence obtaining higher methane coefficients. Biodegradability is also superior during co-digestion, with an improvement of around 10.0% compared to macroalgae anaerobic digestion [11].

Marine biomass has reported improved outcomes over terrestrial energy crops for stable methane production [34]. Indeed, data established anaerobic co-digestion as more effective both in terms of methane yield and biodegradability values than mono-digestion [11].

#### 3.1.2. Composting

**Figure 5:**
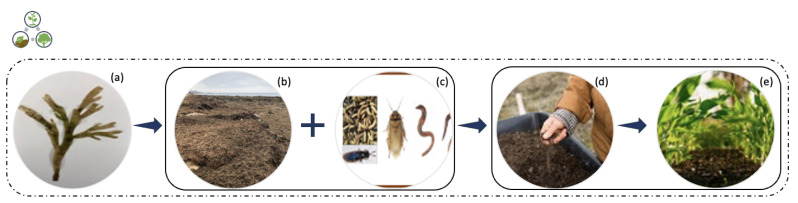
Composting process using *R. okamurae* biomass. (**a**) Photograph of *R. okamurae* alga collected from the coast of São Miguel Island. (**b**) *Rugulopteryx okamurae*, invasive seaweed of on the coast of the Strait of Gibraltar (adapted from [10], published by Springer in 2022 with CC BY 4.0 permission). (**c**) Invertebrate species studied in [10] (adapted from [10], published by Springer in 2022 with CC BY 4.0 permission). (**d**) Photo by Zoe Schaeffer by Unsplash License in 2021, (**e**) Plantation, photo by Steven Weeks by Unsplash License in 2021.

Composting is an aerobic process (Figure 5) in which organic matter is degraded by the activities of microorganisms. It is an old approach that has been gaining more attention and significance in recent years [10,36]. It is a process of aerobic biotransformation of organic matter, through which a product known as compost is obtained. This final product has a high bio-fertilizing character, among other agronomic benefits [37].

Invasive marine algae biomass has been used for composting and transformed into agricultural products [10], with excellent bio-fertilizing profiles [37]. The composting quality is intimately dependent on the raw materials properties, preparation methods and maturation time [36]. Some marine algae species have high salt content, metal supplements or low carbon/nitrogen ratios. Therefore, microbiological supplements (i.e., forest residues with high carbon levels, small amounts of fishery, etc.) are added to the compost to compensate the deficiencies found.

*R. okamurae* has been identified as highly toxic due to its high concentration in sesquiterpenes, not found in other algae. This particularity of *R. okamurae* makes it impossible to apply the most traditional composting technique, the vermicomposting. Additionally, because of these algae’s high terpene contents (largest fraction of volatile organic compounds, VOC), it is not easily degraded. Thus, Patón et al. [10] proposed to test the bioremediation method with five different invertebrates (vermicomposting (*Dendrobaena veneta* and *Eisenia fetida*), blatticomposting (*Eublaberus* spp. “Ivory”), mealworms (*Tenebrio molitor*) and black soldier fly larvae (BSFL, *Hermetia illucens*)) to identify whether invertebrate species could resist the high concentration of terpenes in this species. Their results suggested that composting made with BSFL and with cockroaches used to convert human food wastes into composting (blatticomposting) were highly effective, as macroalgae toxicity did not affect the long-term survival, growth and reproduction of the invertebrates. The success of this study attested to the use of these invasive species biomass as composting, this way facilitating access to cheaper bio-fertilizers and animal proteins for municipalities located in the affected regions. Still, it should be noticed that the invasive algae are not compostable when pure. However, if combined with conditioning agents such as leftovers of garden or vegetables, they can be processed through conventional composting approaches [10].

In another study, a comparative analysis of bio-fertilizers with different proportions (by volume) of the invasive algae was carried out: 100% algae, 66% vegetable waste and 33% algae, and 66% pruning residues and 33% algae. Data deemed *R. okamurae* composting as very promising particularly in combination with vegetable wastes, this way enabling the development of agronomic products with great environmental impact within the circular economy model [37].

#### 3.1.3. Bioplastics

Bioplastics are degradable materials, of which applications are very similar to those of plastics [38]. Macroalgae can be considered sources for bioplastic production due to their low cost, low toxicity and suitable mechanical properties [39].

Marine algae are rich in polysaccharides, such as carrageenan, agar and alginate, which are great materials for producing bioplastics. Up to 40% of the dry weight of the brown algae is made of alginates [29,38]. Alginate is an anionic linear polysaccharide, composed of monomeric units of β-D-mannuronic acid (M) and α-L-guluronic acid (G) joined by 1,4-glycosidic bonds. The physicochemical and mechanical properties of alginate gels depend on the M/G ratio and polymeric structure length. Alginates are very hydrophilic; hence, to ensure water resistance, other elements are frequently combined with the alginate. Another option is to form bonds with cations. The addition of calcium ions (i.e., $CaCl_{2}$) to the alginate matrix increases the stability and resistance of the formed film/hydrogel [29].

The potential of the algae *R. okamurae* as a raw material for bioplastic uses was analyzed by Santana et al. [39]. The study referred to the mixture of seaweed (RO) with glycerol (GLY) according to the RO/GLY ratio in the following proportions: 50/50, 60/40 and 70/30%. The process and production of the bioplastics started with the lyophilized and ground algae, which were then mixed with glycerol through a Haake polylab QC mixer (ThermoHaake, Karlsruhe, Germany). Data reported the potential of the invasive algae to produce bioplastics via injection molding, using glycerol as a plasticizer. Researchers have reported that the higher the content of seaweed, the greater the viscoelastic properties, rigidity and resistance of the obtained bioplastics. Through dynamic mechanical testing, they also demonstrated the algae’s typical thermoplastic behavior, with the prevalence of an elastic profile in detriment of a viscous. Researchers observed that the higher the biomass content, the lower the water uptake capacity as it reduced the hydrophilic character of the used plasticizer. It was also noted that the mechanical stability of the material and water absorption capacity are inversely proportional since the polymers arranged dimensionally in cross-linked networks prevented more water molecules from entering the porous structure and bond. This also had an impact on reducing the soluble matter loss. In the tensile test, the mold temperature variation was more noticeable, as the system molded at 120 ºC stood out slightly since a small reduction in water absorption capacity was detected with increasing mold temperature. The results confirmed *R. okamurae* as a promising source of bioplastic raw matter but identified the mechanical deficiencies of the algae to serve as base substrate in a bioplastic. Composite formulations may be an effective way to overcome such limitations [39].

### 3.2. Pharmacological Applications of R. okamurae

#### 3.2.1. Anti-Inflammatory Activity

The search for new anti-inflammatory agents is of great relevance to the medical field since inflammation is associated with several difficult to treat diseases, such as some types of cancer, rheumatoid arthritis, inflammatory bowel disease and diabetes [12].

Brown algae belonging to the Phaeophyceae class, such as *R. okamurae*, are formed of large amounts of terpenoids, most of which diterpenes, sesquiterpenoids and meroditerpenes [40]. Some species, including *R. okamurae*, are characterized by producing a wide range of cyclic diterpenoids. These metabolites have a variety of carbon skeletons, which differ significantly between genera and can be useful chemotaxonomic markers [12]. They are composed of five carbon subunits (isoprenoids) arranged in a configuration called head and tail and are classified according to the number of isoprenoid units incorporated [41]. Most of the research that addresses terpenoids isolated from this alga is focused on their ecological function as feeding deterrents of predators. Few data have been analyzed for their biomedical potential. Until recently, only the antibacterial activities of some secospatanes (diterpene class) against *Bacillus subtilis* were reported [12].

**Figure 6:**
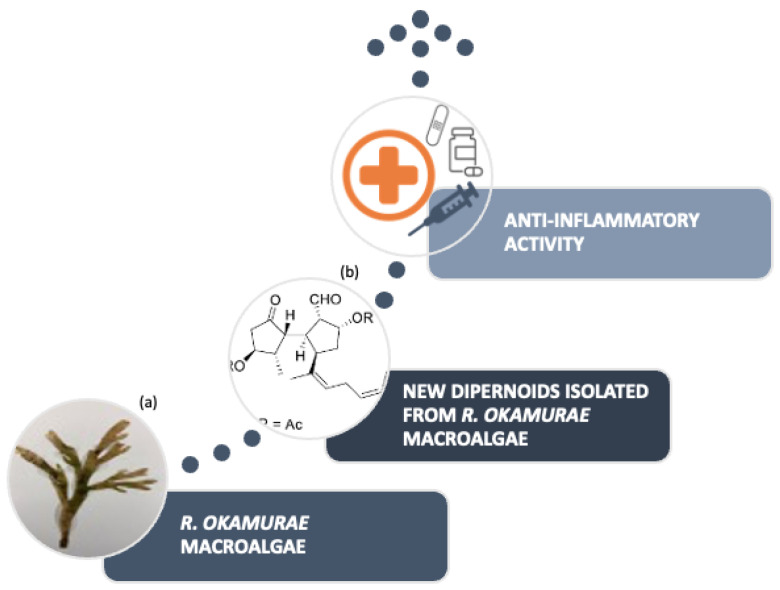
Investigation process using extract of *R. okamurae* (**a**) Photograph of *R. okamurae* alga collected from the coast of São Miguel Island. (**b**) New Diterpenoids Isolated from *R. okamurae* (adapted from [12], published by MDPI in 2021 with CC BY 4.0 permission).

Research conducted (Figure 6) on the algae species located in the Strait of Gibraltar allowed for the isolation of six ditepernoids of the secospatane, spartan and prenylcubebane types.

**Figure 7:**
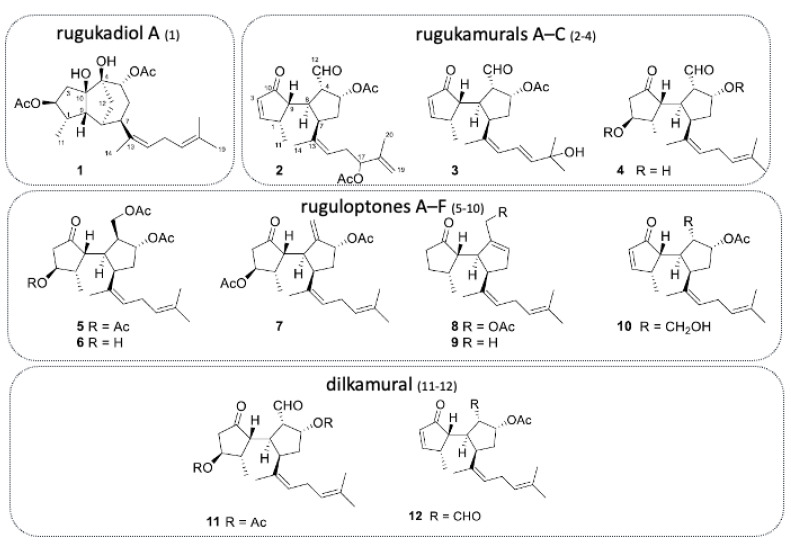
Chemical structures of diterpenoids isolated from *R. okamurae* (adapted from [12], published by MDPI in 2022 with CC BY 4.0 permission).

More recently, the isolation of ten new diterpenoids has been successfully accomplished: rugukadiol A (**1**), rugukamurals A–C (**2**–**4**) and ruguloptones A–F (**5**–**10**). In addition, compounds already known such as dilkamural **11** and **12**, which are the main metabolites of the extract, were obtained (Figure 7).

The isolated compounds were tested in an attempt to detect inhibition in the production of the inflammatory mediating agent nitric oxide (NO) and the expression of Nos2 and pro-inflammatory cytokines. Anti-inflammatory assays were performed on Bv.2 immune cells (microglia) and RAW 264.7 cells (macrophages), important mediating cells in inflammatory processes. The method consisted in stimulating these immune cells by bacterial compounds, such as lipopolysaccharide (LPS), which cause the synthesis and release of NO and pro-inflammatory cytokines [12].

The experiment started with the verification of the cytotoxicity of different concentrations of the compounds for Bv.2 and RAW 264.7 cells. The obtained results (in concentrations equal to or lower than 10 µM) were as follows: compounds **1**, **4**, **5**, **6** and **10** did not show cytotoxic effects for both cells; compound **4** showed a cytotoxic effect on macrophages; and compound **7** showed a cytotoxic effect on Bv.2 and RAW 264.7. Compounds **11** (dilkamural) and compound **12** exhibited significant cytotoxicity at 10 µM against Bv.2 cells. Compounds **2**, **3**, **8** and **9** were discarded for assays; compounds **2** and **8** were discarded due to a lack of material; compound **9** was discarded due to solubility problems; and compound **3** was discarded because it is analogous to compound **12** [12].

Then, the effect of non-cytotoxic compounds **1**, **4**, **5**, **6** and **10** on NO production was verified, in which the cells were pre-treated with the compounds and then stimulated with LPS. Nitrite levels in Bv.2 and RAW 264.7 cells treated with compounds but not stimulated did not change relative to non-treated cells [12].

After treatment of Bv.2 control cells with LPS, the level of nitrites increased significantly. However, in cells pre-treated for 3 h with compounds **1**, **4**, **5**, **6** or **10**, LPS-stimulated nitrite production was significantly inhibited. Compounds **1**, **5** and **10** were considerably active, resulting, in 10 µM, 68.1%, 70.0%, and 60.0% inhibition of nitrite production, respectively, compared to stimulated in untreated cells. The inhibitory effects of **1** and **5** were greater than those produced by the reference compound dexamethasone at 2.5 µM. Compounds **4** and **6** were less active, causing 48.1% and 46.0% inhibition, respectively [12].

Assays with RAW 264.7 cells showed a similar result to assays with Bv.2 cells. Cells stimulated with LPS significantly increased nitrite content. Pre-treatment with compounds **1**, **5** and **10** at 10 µM inhibited LPS-stimulated nitrite production by 63.2%, 69.2%, and 64.9%, respectively. These effects were slightly less than those generated by 2.5 µM dexamethasone. Compound **6** was again the least active, causing 56.6% inhibition of nitrite production [12].

Regarding the results referring to compounds **11** and **12** against Bv.2 cells, compound **11** did not have a significant effect on NO production, and compound **12** caused 30% inhibition and in assays with RAW 264.7 cells. Cell treatments with LPS significantly increased nitrite content [12].

The results uncovered the anti-inflammatory potential of the diterpenoids rugukadiol A (**1**), ruguloptone A (**5**) and ruguloptone F (**10**). They were able to almost completely neutralize the effects of LPS stimulation on cells, keeping NO concentrations close to basal levels (unstimulated cells). Additionally, among the secospatanes tested, they identified a possible correlation between the presence of a primary hydroxy or acetoxy group at C-12 with anti-inflammatory activity [12].

With the intention of obtaining more data on the anti-inflammatory effects, assays were carried out to analyze the inhibition of Nos2 expression and pro-inflammatory cytokines in compounds **1** and **5**, which showed greater potential activity of inhibitory activity on NO secretion. After stimulation with LPS, significant increases in Nos2 and Il1b mRNA expression were detected in Bv.2 cells [12].

This research was the first report on anti-inflammatory activity within the secospatane class of metabolites. In addition, it made reference to a new structural class represented by compound **1**, extending the range of biological sources and structural variety of marine diterpenoids with anti-inflammatory potential. This investigation revealed that *R. okamurae* contains a variety of compounds, with possible interest for pharmacological purposes in the anti-inflammatory area. It is also worth noting that seaweed taken from the Strait of Gibraltar contains high concentrations of the diterpene dilkamural **11** and of its elimination product **12**. These compounds may be discarded for anti-inflammatory studies but may have important properties in other therapeutic areas [12].

#### 3.2.2. Antibacterial Activity

**Figure 8:**
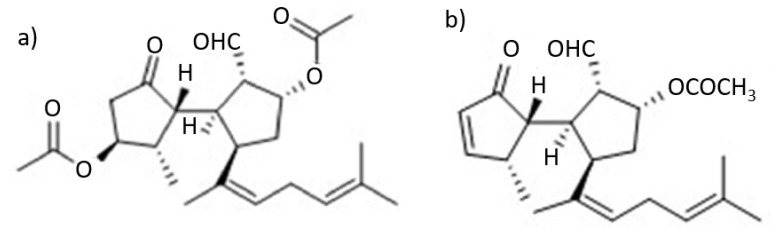
(**a**) Chemical structure of secospatane diterpene isolated (dilkamural) from *D. okamurae* collected in Shikoku Island of Japan (adapted from [42], published by SAGE Journals in 2020 with CC BY 4.0 permission). (**b**) Chemical structure of Secospatane Diterpene isolated from *D. okamurae* collected in Collaroy, New South Wales of Australian (adapted from [42], published by SAGE Journals in 2020 with CC BY 4.0 permission).

As previously mentioned, marine organisms have unique secondary metabolites with important biological properties and pharmaceutical activities. The brown macroalgae *R. okamurae* (previously designated as *Dilophus okamurae*) was collected for investigation from Japan’s Shikoku Island, at the intertidal zones around the Honai Beach, Ehime Prefecture, Japan in May 1994. The investigation was focused on the bioactive compound secospatane-type diterpenoid 1, also known as dilkamural (Figure 8a). The isolated compound was identified by NMR [25]. The bio-compost was extracted from the algae in the form of a colorless oil with a yield of 1.9% of the total dry mass. The molecular formula of $C_{24}H_{34}O_{6}$ was established by high resolution mass spectrometry [25].

The metabolite dilkamural and another secospatane diterpene (Figure 8b) obtained from the brown alga *D. okamurae* collected in Collaroy, New South Wales of Australian showed moderate antimicrobial activity against the Gram-positive bacterium *Bacillus subtilis*, by revealing 12 and 9 mm, respectively, of inhibitory zone diameters via agar-disk diffusion method (10 µg/disk). The inhibitory activity results were considered weak [25]. Additionally, dilkamural may be effective against pathogenic microorganisms present in new habitats [24].

Considering the lack of information about this alga antimicrobial profile, there is a great opportunity for pioneer investigation to be made in the next few years.

#### 3.2.3. Inhibition of α-Glucosidase

Macroalgae have been considered great sources of natural extracts with potential for antidiabetic formulations [43,44,45] as a way to replace existing commercial α-glucosidase inhibitors, which cause adverse reactions, namely severe gastrointestinal disorders. Therefore, there is currently a great interest from researchers in finding any natural safe and effective α-glucosidase inhibitor capable of replacing others such as acarbose and voglibose [45].

α-Glycosidases are exo-acting carbohydrase enzymes that catalyze the release of α-D-glucopyranose from the non-reducing ends of various carbohydrate substrates [45,46]. α-Glucosidase inhibitors function as oral antihyperglycemic agents, delaying intestinal absorption of carbohydrates and decreasing the postprandial rise in glucose levels [45,47]. Researchers believe that α-glucosidase inhibitors may be useful in treatments for carbohydrate-mediated diseases, such as diabetes and obesity.

*R. okamurae* extracts have been quantified for their α-glucosidase inhibitory functions by their enzymatic activity. The extracts were tested in comparison with acarbose (used as a positive control) demonstrating their potential in inhibiting 90.9% of the α-glucosidase activity compared to the 70.3% attained by acarbose. In this context, *R. okamurae* has been suggested as a promising component for new diabetes drugs or for physiologically functional foods and pharmaceuticals [45].

## 4. Conclusions and Future Perspectives

This work reported on the existing and potential uses of the algae *R. okamurae*, highlighting possible ways to minimize the effect of the large amount of biomass accumulated in Europe waters and the Strait of Gibraltar. The applications found were biomass use for biogas production, composting, development of bioplastic material, and pharmaceutical formulations with anti-inflammatory and antimicrobial activities or α-glucosidase inhibitory functions. Applications based on the algae *R. okamurae* can help reduce costs related to the collection of the macroalgae carried out by municipalities in the affected regions. In addition, it is a way of giving value to a waste that is frequently accumulated in landfills.

Research on the use of this species as a substrate in aerobic digestion and composting revealed its inability to work on its own for such purposes but its ability to be performed at optimal ranges as a co-substrate. The alga, when combined with a co-substrate rich in nitrogen, instigates biodegradation of organic matter. Therefore, the use of *R. okamurae* as a co-substrate can be a means of eliminating the biomass of invasive species and a relevant alternative to minimize such a problem to many ecosystems. In addition, the use of biomass with a focus on product development can be explored in other areas of study, such as textiles, which research has not yet been reported.

Currently, defining the antimicrobial potential of this alga is a considerable challenge. Even though antimicrobial activity has been identified against one bacterium, a more detailed and in-depth investigation of the high-spectrum antimicrobial properties of *R. okamurae* is still lacking and therefore highly recommended.

Investigations that sought to identify the anti-inflammatory and antimicrobial activities and the potential of *R. okamurae* as an antidiabetic functional food have already taken the first steps but are still incipient and require further exploration. Additional studies should comprehensively be conducted to discover the metabolites and the real contribution of *R. okamurae* components for medicinal purposes.

In addition, studies on antidiabetic functional food should be further explored, with a focus on improving its effectiveness for new drug formulations applications.

Once these bioactive profiles are proven, this alga may find future applications as well in the cosmeceutical area via the development of bioproducts beneficial to skin care, or within the medical textiles industry (i.e., wound dressings). Similarly, research on the use of *R. okamurae* as a basis for the development of a bioplastic material requires further investigations to improve the mechanical properties of the algae-based bioplastic engineered.

The increased development of natural products based on brown macroalgae of the same class as *R. okamurae* encourages new discoveries and motivates the application of this alga biomass as additive or main material in new product formulations.

In this study, there was a limited number of investigations on the applications of *R. okamurae*, and therefore, there is an opportunity for future investigations to be carried out with the use of this alga; perhaps by using the same principles and exploration techniques employed with other brown algae.

Investigations into applications of seaweed extracts from brown seaweeds of the Dictyotaceae family are being driven by increased interest in their secondary metabolites, mainly diterpenes. Of the genera of the Dictyotaceae family, *Dictyota* has been noted due to the several secondary metabolites identified (e.g., several phenols and terpenoids, mainly the diterpenes) [48]. A study made with seaweed collected in Tateyama Bay, Japan, of the *Dilophus okamurae* specie, formerly known as *R. okamurae*, verified a similarity in terms of morphology between *D. okamurae* and the brown seaweed species of the genus *Dictyota* [49]. Therefore, some existing applications of *Dictyota* will be presented below, as a stimulus for the prospective application of biologically active compounds discovered and extracted from *R. okamurae*.

In the pharmaceutical area, research using extracts from species of the genus *Dictyota* revealed a possible neuroprotective effect for the treatment of Alzheimer’s disease. The identified methanolyte extract was found to possess an inhibitory effect against the enzyme butyrylcholinesterase (BuChE), responsible for correct cholinergic deficit [48,50]. Another extract of scientific interest is the heterofucans, which possesses a strong anticoagulant activity that can help in the treatment of cardiovascular pathologies, such as dysfunction in coagulation and platelet aggregation [48,51,52]. Extracts from brown algae species of the genus *Dictyota* have also demonstrated possible anticancer, antiviral, antifungal, antileishmanial, antitrypanosomal and antioxidant activities [48]. Another important discovery is associated with the display of antiviral activity against Herpes Simplex Virus and Human Immunodeficiency Virus [53].

As a contribution to the cosmeceutical field, the discovery of the diterpene compound from the *D. coriacea* species revealed positive anti-melanogenesis effects to be used in case of excess melanin production, which can cause hyperpigmentation in the skin such as freckles and age spots [48,54]. Dermal applications of the genus *Dictyota* have already been reported in investigations, such as their potential for the treatment of alopecia, but until now, there has been no reports of these compounds being used in the production of cosmetics [53].

In the textile industry, *Dictyota* also plays a role in fabric engineering [53,55], normally used as a binding or thickening material for yarns, promptly reducing yarn tearing during the weaving process [55].

It is expected that the interest in investigations on *R. okamurae* with regard to bioactivity and its properties will be boosted with the possibilities of investigations presented on algae of the genus *Dictyota* and their significant results in the scientific field.

## Figures and Tables

**Table 1:** Compounds isolated per diethyl ether extract (%) from *R. okamurae* collected at the Strait of Gibraltar [24].

Isolated Compounds	Extract (% *w*/*w*)
Compound **1**—dilkamural	28.25
Compound **2**	3.17
Compound **3**	0.23
Compound **4**	0.21
Compound **5**	0.17
Compound **6**	1.55

## Data Availability

Not applicable.
